# Insights into the Molecular Basis of L-Form Formation and Survival in *Escherichia coli*

**DOI:** 10.1371/journal.pone.0007316

**Published:** 2009-10-06

**Authors:** William A. Glover, Yanqin Yang, Ying Zhang

**Affiliations:** 1 Department of Molecular Microbiology and Immunology, Bloomberg School of Public Health, Johns Hopkins University, Baltimore, Maryland, United States of America; 2 Bioinformatics Core, The Wilmer Eye Institute, School of Medicine, Johns Hopkins University, Baltimore, Maryland, United States of America; University of Hyderabad, India

## Abstract

L-forms have been shown to occur among many species of bacteria and are suspected to be involved in persistent infections. Since their discovery in 1935, numerous studies characterizing L-form morphology, growth, and pathogenic potential have been conducted. However, the molecular mechanisms underlying the formation and survival of L-forms remain unknown. Using unstable L-form colonies of *Escherichia coli* as a model, we performed genome-wide transcriptome analysis and screened a deletion mutant library to study the molecular mechanisms involved in formation and survival of L-forms. Microarray analysis of L-form versus classical colonies revealed many up-regulated genes of unknown function as well as multiple over-expressed stress pathways shared in common with persister cells and biofilms. Mutant screens identified three groups of mutants which displayed varying degrees of defects in L-form colony formation. Group 1 mutants, which showed the strongest defect in L-form colony formation, belonged to pathways involved in cell envelope stress, DNA repair, iron homeostasis, outer membrane biogenesis, and drug efflux/ABC transporters. Four (Group 1) mutants, *rcsB*, a positive response regulator of colanic acid capsule synthesis, *ruvA*, a recombinational junction binding protein, *fur*, a ferric uptake regulator and *smpA* a small membrane lipoprotein were selected for complementation. Complementation of the mutants using a high-copy overexpression vector failed, while utilization of a low-copy inducible vector successfully restored L-form formation. This work represents the first systematic genetic evaluation of genes and pathways involved in the formation and survival of unstable L-form bacteria. Our findings provide new insights into the molecular mechanisms underlying L-form formation and survival and have implications for understanding the emergence of antibiotic resistance, bacterial persistence and latent infections and designing novel drugs and vaccines.

## Introduction

Bacteria can be found in every niche on earth. Within these niches, bacteria exist in an array of sizes and morphologies. An important contributor to this diversity of cellular morphologies is the bacterial cell wall. The cell wall is not only important for maintaining cell shape but also for protection in constantly changing environments. Under certain conditions bacteria can spontaneously or by induction, lose part or all of their cell wall [1]–[3], resulting in osmosensitive cells known as cell wall deficient or defective bacteria (CWDB). CWDB can be generated *in vitro* and *in vivo* among many species of bacteria, and therefore represent a plausible survival strategy utilized by bacteria to escape killing by cell wall targeting antibiotics and the immune system [4]. CWDB capable of “fried egg” growth on specialized solid media are termed L-forms, which can be classified into four groups [5] based upon their ability to remain in the L-form state (unstable versus stable L-forms) and the presence or absence of residual cell wall (spheroplast-type versus protoplast-type L-forms). Many aspects of L-form manipulations can be carried out in liquid media; however, L-forms are best verified by observing growth on specialized solid media which often resemble “fried egg” colonies exhibited by mycoplasmas [6].

Since their discovery by Emmy Klieneberger in 1935 [7], L-forms, named in honor of the Lister Institute where she worked, have been suspected to be causative agents of disease underlying chronic and persistent infections [4], [6], [8]. Although numerous publications exist characterizing their morphologies, growth requirements, and isolation from humans and animals with chronic infections [4], [9]–[11], the role of L-forms in disease has been difficult to ascertain. This, in part, is due to lack of understanding of the basic biology of L-forms and the circumstances favoring the transition of classical bacteria into L-forms. Additionally, the fact that L-forms can be obtained *in vitro* by multiple methods which can influence subsequent analyses and that multiple types of L-forms appear to exist, have made the design of a definitive study implicating these forms as causative agents extremely challenging. More importantly, difficulty in establishing standardization within the field has contributed to the lack of clarity in the field, which ultimately led to neglect and abandonment of research on these forms [5] until recently.

A renewed interest in L-form research has emerged as of late [12]–[16]. In 2005, Fuller et al. conducted research on the osmotic stability of unstable *Staphylococcus aureus* L-forms and inherited beta-lactam resistance in revertants. In 2006, Siddiqui et al. analyzed the *dcw* locus and identified mutations in the stable *E. coli* L-form strain LW1665F^+^. Joseleau-Petit et al. studied cell division and peptidoglycan synthesis of unstable *E. coli* L-forms and identified regulatory and structural colanic acid mutants and an *mrcB* penicillin-binding protein (PBP) 1B mutant defective in L-form formation, in 2007. More recently, Leaver et al. examined *ftsZ* independent cell division and an *ispA* mutation in stable *Bacillus subtilis* L-forms, and Dell'Era et al. observed cell division and changes in gene expression in stable *Listeria monocytogenes* L-forms. With the exception of Joseleau et al. much of this renewed research has been conducted on Gram-positive bacteria and/or stable L-forms which usually required mutagenesis or long-term passages for generation. Historically, it has been known that stable L-forms are genetically different from their parent strain due to accumulation of mutations during the lengthy process of their selection, although the mutations involved remain poorly characterized [5], [17]. Stable L-forms represent good models to study basic biological functions in L-forms, their ability to cause disease, and mechanisms involved in their inability to revert to classical bacteria. However, they are considered to be distinct entities from unstable L-forms although they appear to share commonalities. Unstable L-forms are considered to be genetically identical [5] to their parent strain and retain their ability to revert back to the classical form. Thus, unstable L-forms represent a unique opportunity to examine the molecular processes underlying the transition of classical bacteria to the L-form phenotype and the gene expression alterations that occur as a result of this transition.

In this study, we first established an unstable *E. coli* L-form model and then conducted genome-wide transcriptome analysis comparing classical colonies and antibiotic induced unstable L-form colonies to investigate changes in gene expression that result from transition to L-forms. Microarray analysis revealed drastic changes in the transcription profile of *E. coli* L-forms, which included the overexpression of many genes involved in stress responses and of unknown function that may be involved in the formation and survival of L-forms. Using an optimized L-form induction media, we screened an *E. coli* deletion mutant library for mutants defective in forming L-form colonies. This approach successfully identified various mutants belonging to cell envelope stress, DNA repair, iron homeostasis pathways, outer membrane biogenesis, and drug efflux/ABC transporters. Four mutants, *rcsB*, a response regulator of colanic acid capsule synthesis, *ruvA*, a recombinational junction binding protein, *fur*, a ferric uptake regulator, and *smpA*, a small membrane lipoprotein, were chosen for complementation studies and all were found to be required for L-form colony formation. These results have implications for understanding the emergence of antibiotic resistance, bacterial persistence and latent infections and designing novel drugs and vaccines.

## Results

### Generation of antibiotic induced unstable *E. coli* L-forms

L-form induction media (LIM), a Brain Heart Infusion based media, described by Huber and Brinkley was tested for its ability to induce strains of *E. coli* K-12 (W3110 and BW25113) to grow as L-form colonies [18]. Log phase and stationary phase cells of both strains produced L-form colonies when plated directly onto LIM. Increase of penicillin G (Pen G) concentration from 600 µg (1000 units)/ml to 6000 µg (10,000 units)/ml in LIM did not appear to affect the ability of the bacteria to form L-form colonies, but increased the reversion time of L-form colonies to classical colonies from 2 days to 5 days. The minimum bacterial inoculum required for L-form colony formation was approximately 10^4^–10^5^ bacterial cells, which is consistent with the finding reported previously in the literature [12].

**Figure 1:**
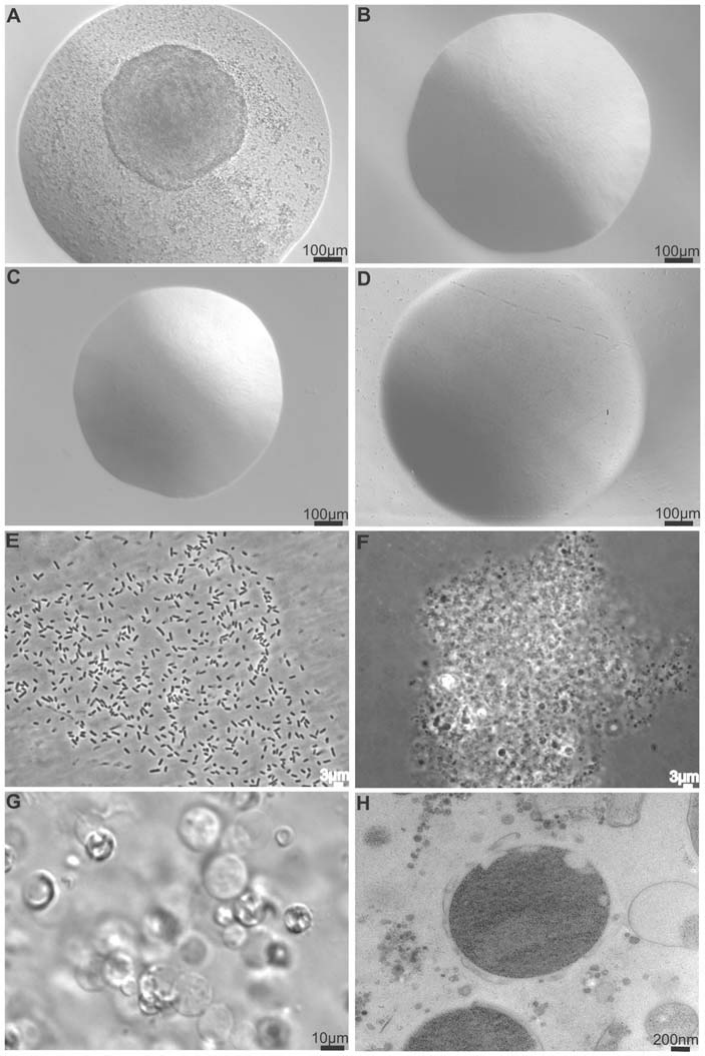
Comparison of L-form *E. coli* and classical *E. coli* morphologies. A *E. coli* colony on L-form induction media (LIM) exhibiting typical “fried egg” morphology. B Classical *E. coli* colony on Brain Heart Infusion (BHI) agar. C *E. coli* colony on BHI+ 10% Sucrose and 0.125% MgSO4 (BHI+ sucrose control media). D *E. coli* penicillin G mutant colony on BHI+ Pen G. E Phase contrast of rod-shaped *E. coli* cells within a classical colony. Scale bar, 3 µm. F Phase contrast of coccoid cells within *E. coli* L-form colony agar squash. Scale bar, 3 µm. G Individual coccoid cells in soft agar LIM. Scale bar, 10 µm. H Transmission electron microscopy (TEM) photo of a coccoid cell within an L-form colony. Scale bar, 200 nm. All images are by Hoffman modulation and scale bars are 100 µm unless specified.

*E. coli* L-form colonies appeared in 48–72 hrs, were mucoid, and exhibited typical “fried egg” morphology, consisting of peripheral growth on the surface of the agar with a dense center embedded into the agar (**Fig. 1A**). In contrast, classical colonies grew overnight, were homogeneous, and appeared smooth on the surface of BHI (**Fig. 1B**) and BHI+sucrose control media (**Fig. 1C**). Penicillin G resistant mutant colonies grew in 48 hrs and also appeared as smooth and homogenous classical colonies on BHI+penicillin G (**Fig. 1D**). Compared to rod-shaped cells within classical colonies (**Fig. 1E**), microscopic observation of an agar-squashed L-form colony revealed numerous tightly packed coccoid cells (**Fig. 1F**). Subculture of the coccoid cells into fresh LIM showed aggregation and proliferation resulting in growth of new L-form colonies (**Fig. 1G**). Freeze-substitution transmission electron microscopy of an L-form colony revealed heterogeneous coccoid cells of varying shapes and sizes. Cells also varied with respect to the presence or absence of cell wall (**Fig. 1H**). L-form colonies of both *E. coli* strains tested were stable for at least 5 days, but reverted to classical colonies to varying degrees upon extended incubation. Therefore, we used newly induced L-form colonies formed within 72 hrs for our subsequent molecular studies.

### Gene expression changes in *E. coli* L-form colonies

In order to examine gene expression changes that occur as a result of conversion into L-form colonies, we performed microarray experiments utilizing *E. coli* 2.0 Genome Genechip arrays (Affymetrix). *E. coli* colonies grown under three conditions BHI (BHI control), BHI+sucrose+MgSO_4_ (sucrose control), and BHI+sucrose+MgSO_4_+penicillin G (L-form) were harvested and analyzed in triplicate. *E. coli* K-12 strain W3110 cells grown within 24 hr classical colonies on BHI+ sucrose+ MgSO_4_ as a control, were compared to cells grown within 72 hr L-form colonies on BHI+sucrose+MgSO_4_+Pen G. This ensured that colonies were similar in size and phase of growth since L-form colonies grow slower than classical *E. coli* colonies.

**Figure 2:**
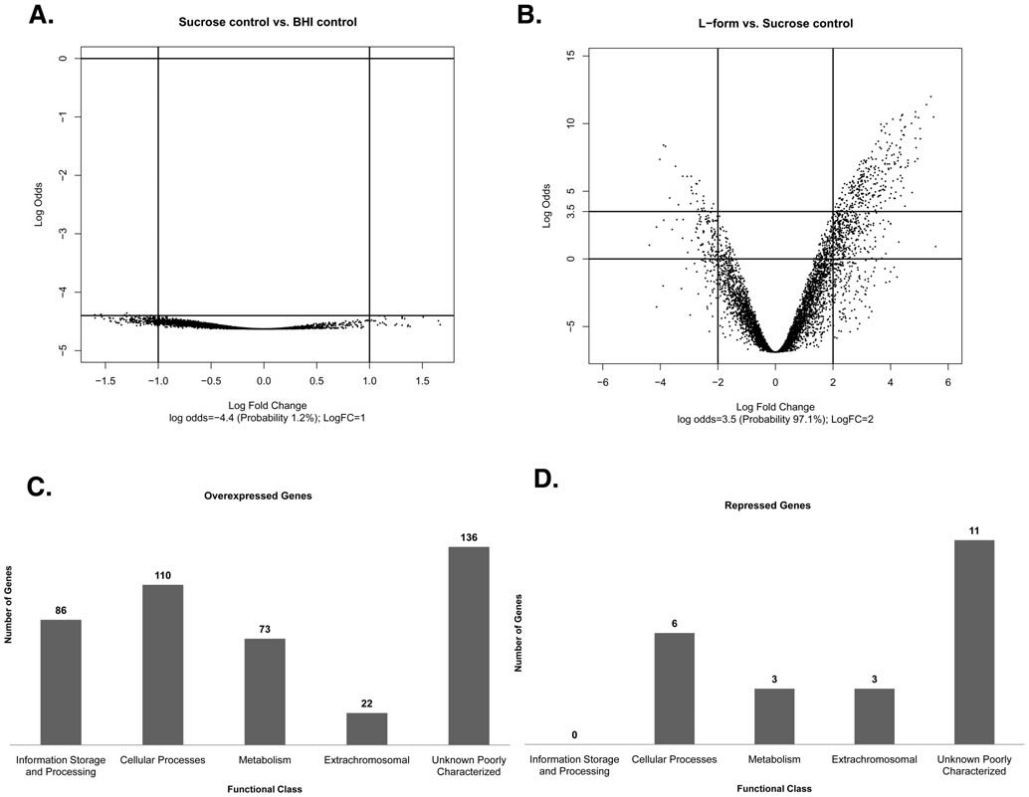
Graphical display of *E. coli* L-form microarray analysis. A Volcano plot of log-fold changes versus log-odds of differential expression comparing BHI sucrose control to BHI control arrays. Each dot represents one gene on the plot. A log odds value of 0 (horizontal line) in each graph corresponds to a 50% chance that the gene is differentially expressed. The cut-off log-odds value at −4.4 (1.2% probability) indicates there are no significant differentially expressed genes between these two conditions. B Comparison of L-form versus Sucrose control arrays showing a fourfold change cut-off ratio and a 3.5 (97.1% probability) log-odds value. C Breakdown of the 427 overexpressed genes into 5 functional classes. D Schematic of the 23 repressed genes categorized into 4 functional classes.

Identification of significant differentially expressed genes was determined using the Linear Model for Microarray Data (Limma) package in Bioconductor [19]. No significant differential gene expression was found between BHI+sucrose control colonies and BHI control colonies (**Fig. 2A**). Comparison of L-form colonies versus BHI+sucrose colonies (**Fig. 2B**) yielded a large number of differentially expressed genes, therefore a fourfold cut-off ratio and a 3.5 log-odds value was applied to the data. Application of both filters resulted in 450 significant genes with a probability of 97.1% of being differentially expressed equal to or greater than fourfold. Of these 450 genes, 427genes (94.9%) were up-regulated and 23 genes (5.1%) were down-regulated (**Fig. 2B** **, Table S1, S2**).

The breakdown of significant differentially overexpressed genes in L-form colonies into 5 main functional classes using the COG functional classification system (**Fig. 2C** **, Table S1**) showed that the two classes containing the most genes were involved in unknown or poorly characterized functions (31.8%) and cellular processes (25.8%). The remaining functional classes of overexpressed genes included those involved in information storage and processing (20.1%), metabolism (17.1%), and extrachromosomal elements (5.2%) (**Fig. 2C**). Significant differentially repressed genes could be divided into 4 main functional classes (**Fig. 2D** **, Table S2**). Approximately half (48%) of the repressed genes were involved in unknown or poorly characterized functions. Enriched pathways among overexpressed genes consisted of numerous stress responses such as DNA repair/SOS response, heat shock, phage shock, and envelope stress. Other significant enriched pathways included: sulfate assimilation and cysteine biosynthesis, iron sulfur cluster repair and biogenesis, and phosphate uptake (**Table S1**). Many genes involved in lipopolysaccharide core biosynthesis, two component systems, toxin-antitoxins modules, osmoregulation, ABC transporters/drug efflux pumps, intracellular signaling, and numerous transcription factors, were up-regulated as well. Also of note is the fact that several small RNAs, and two genes involved in motility and chemotaxis were enriched among the few down-regulated genes (**Table S2**). The remainder of this paper focuses on genes which were overexpressed in L-form colonies, the downregulated genes will be addressed at a later time. Following the identification of enriched pathways, real-time PCR confirmation was performed on 11 up-regulated genes among identified pathways. Results of the real-time analysis correlated with our microarray data (**Table S3–S4**).

### Mutant screen to identify genes involved in L-form colony formation

**Figure 3:**
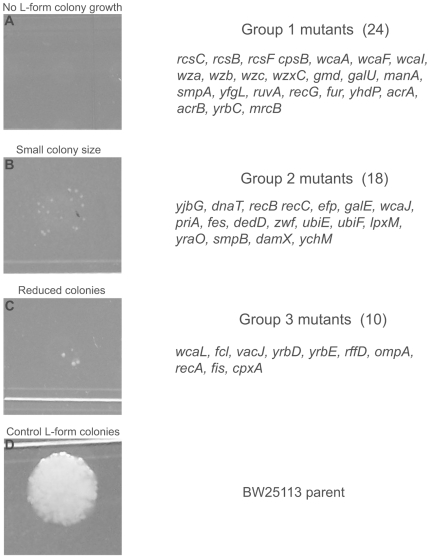
L-form colony phenotypes exhibited during screening of the *E. coli* Keio deletion mutant library. A No growth phenotype (Group 1) displayed by identified mutants on LIM. B Small colony size phenotype (Group 2) displayed by identified mutants on LIM. C Mutants showing reduced colony numbers (Group 3) on LIM. D *E. coli* BW25113 parent displaying numerous mucoid L-form colonies on LIM.

To investigate genes involved in the formation and survival of *E. coli* L-form colonies, we screened 3985 non-essential mutants of the *E. coli* K-12 BW25113 deletion library for defects in L-form colony formation on LIM. We identified 52 mutants that displayed varying degrees of defects in L-form colony formation (**Fig. 3A–C**) when compared to the parent strain (**Fig. 3D**). Mutants could be divided into three phenotypic groups: no growth (Group 1) (**Fig. 3A**), small colony size (Group 2) (**Fig. 3B**), and reduced colony numbers (Group 3) (**Fig. 3C**). Slightly less than half (24 of the 52) of the identified mutants belonged to Group 1, which showed the strongest defect in L-form colony formation. To minimize false-positives due to growth defects we rescreened stationary phase cultures of the 24 (Group 1) mutants. The results of the rescreen confirmed that the lack of L-form growth was not due to growth defects. Group 1 mutants included genes involved in the Rcs phosphorelay two-component system (*rcsB, rcsC, rcsF*), colonic acid biosynthesis (*cpsB, wcaA, wcaI, gmd, galU, manA, wcaF, wza, wzb, wzc, wzxC*), DNA repair/SOS response (*ruvA, recG*), drug efflux pumps and ABC transporters (*acrA, acrB, ydhP, yrbC*), outer membrane lipoproteins (*smpA* and *yfgL*), transcription factor (*fur*) involved in iron homeostasis, and penicillin binding protein 1B (*mrcB*) (**Fig. 3A**). Consistent with our findings, the *rcs* system and the penicillin binding protein 1B, *mrcB* were previously shown to be involved in L-form colony formation in *E. coli* K-12 strain MG1655 utilizing an alternative induction media and protocol [14].

The remaining 28 identified mutants were distributed among 18 mutants (Group 2) (**Fig. 3B**) which exhibited pinpoint L-form colonies smaller than typical wild type L-form colonies and 10 mutants (Group 3) (**Fig. 3C**) that displayed a reduction in the number of L-form colonies on LIM. Several of the Group 2 and Group 3 mutants mapped to the same functional group or pathways as Group 1 mutants. Group 2 and Group 3 mutants that did not overlap with Group 1 mutants mapped to pathways involved in DNA replication, LPS synthesis, energy metabolism, and siderophore hydrolysis suggesting that genes within Group 2 and Group 3 are not important for the transition of classical bacteria into L-form colonies but rather for improved L-form colony growth.

### Restoration of L-form colony formation among Group 1 mutants

**Figure 4:**
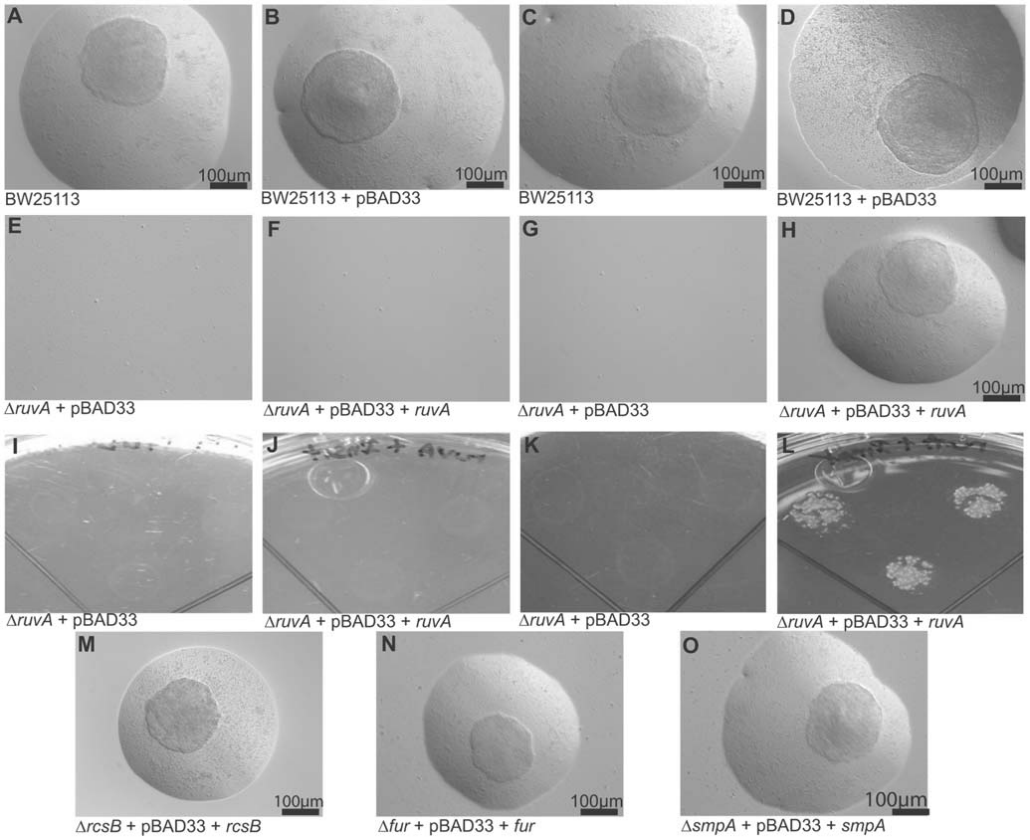
Complementation of deletion mutants restore L-form colony growth. A, B *E. coli* BW25113 alone and BW25113 transformed with empty vector pBAD33 showing “fried egg” morphology on LIM without 0.2% arabinose. C, D *E. coli* BW25113 alone and BW25113 transformed with empty vector pBAD33 showing multiple “fried egg” colonies on LIM with 0.2% arabinose. E, G, I, K *ruvA* mutant transformed with empty vector pBAD33 showing no growth on LIM with and without 0.2% arabinose. F, J *ruvA* mutant complemented with wild type gene showing no growth on LIM without 0.2% arabinose. H, L, M, N, O *ruvA, rcsB, fur,* and *smpA* mutants complemented with their respective wild type genes showing restoration of “fried egg” colony growth on LIM with 0.2% arabinose. All microscopic images are by Hoffman modulation and scale bars are 100 µm unless specified. Images I through L are gross views of LIM plates.

Four Group 1 mutants whose genes were overexpressed in our microarray and showed no growth on LIM were selected for complementation studies to confirm their importance in the formation of L-form colonies. Selected mutants represented pathways involved in colanic acid synthesis (*rcsB*), iron homeostasis (*fur*), DNA repair/SOS response (*ruvA*), and a small lipoprotein component of the outer membrane assembly complex (*smpA*). Complementation of the selected mutants with overexpression high copy number plasmid constructs failed to restore L-form colony formation. Presumably, this was due to toxicity of the overexpressed proteins as well as the vector itself which affected L-form colony formation. Utilizing the arabinose inducible low copy number plasmid vector pBAD33, we successfully complemented all of the selected mutants in their ability to form L-form colonies (**Fig. 4**). The parental strain BW25113 containing the empty vector pBAD33, as well as without the pBAD33 vector, was able to grow as L-form colonies on LIM, with and without (0.2%) arabinose (**Fig. 4A–D**). All mutants containing the empty pBAD33 vector were unable to form L-form colonies in the presence or absence of (0.2%) arabinose, only the *ruvA* mutant is depicted (**Fig. 4E, 4G, 4I, 4K**). All complemented mutants in the absence of (0.2%) arabinose also failed to form L-form colonies (**Fig. 4F, 4J**). Only in the presence of (0.2%) arabinose were the complemented mutants able to form L-form colonies (**Fig. 4H, 4L–O**). These results showed that the four deleted genes in the mutants were indeed responsible for their inability to grow as typical “fried egg” L-form colonies.

## Discussion

**Figure 5:**
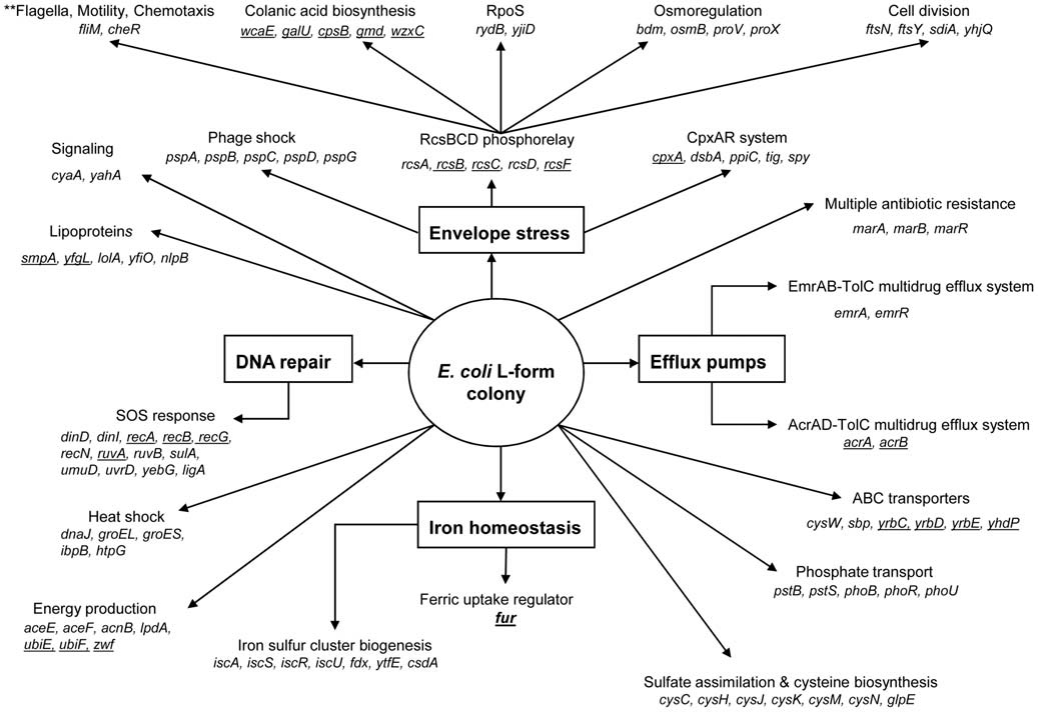
Proposed pathways involved in L-form formation and survival in *E. coli*. Pathways, which are bold and framed, were enriched among Group 1 mutants that failed to form L-form colonies, were overexpressed in our microarray analysis, and were among complemented mutants (except drug efflux pumps). Representative overexpressed genes in the microarray results from different pathways are presented under each pathway. Underlined genes correspond to those that were identified in our mutant screen analysis. Bold underline genes were only present in the mutant screen. ***Represents down-regulated pathway.

Despite the discovery of L-form bacteria in 1935, the molecular mechanisms underlying L-form formation and survival have remained obscure. This lack of progress is mainly because of the unstable nature of L-form bacteria, the variability of the models used for their generation, and the unavailability of modern molecular biology tools before the 1980s when L-form research was largely abandoned. In this study, we took advantage of microarray technology and an *E. coli* deletion mutant library. These tools were used to perform whole genome-wide gene expression analysis and mutant library screens to provide insight into the molecular basis of L-form formation using the *E. coli* L-form as a model. The major findings of this study are the identification of pathways and genes involved in cell envelope stress, DNA repair, iron homeostasis, outer membrane biogenesis, and drug efflux/ABC transporters being involved in L-form formation and survival (**Fig. 5**). This study represents the first systematic genetic analysis of L-form bacteria and provides important insights into the molecular basis of L-form bacteria.

It is of interest to note that our microarray analysis also found enriched overexpressed pathways and genes in unstable L-form colonies that have been described to be overexpressed in *E. coli* persisters, which included phage shock and DNA repair/SOS response pathways along with the TA module MazEF [20], [21]. These findings suggest a relationship between L-forms and persisters may exist and that both combat stress within their environment in a conserved manner. Persister cells may be the surviving cells on LIM which subsequently lead to L-form colony formation. L-form cells could also be a type of persister cell that unlike their walled counterparts, are able to grow in the presence of beta-lactam antibiotics due to the unique conditions in which they are cultured. In addition to persister cells, several enriched overexpressed pathways in L-form colonies were also found to be overexpressed in biofilms, these included stress pathways such as colanic acid, heat shock, DNA repair/SOS, and phage shock [22], [23]. Recently, it was revealed that efflux pumps and transporters are important in biofilm formation [24]. This is consistent with our microarray data which showed up-regulation of AcrAB and EmrAB-TolC drug efflux pumps, as well as the Mar operon. It is highly probable that these multidrug efflux systems contribute to L-form colony formation through removal of toxic substances, including antibiotics. Besides enriched stress pathways, several genes that have been described to be involved in biofilms formation were also among induced genes in L-form colonies. These include *yahA* which encodes a phosphodiesterase with specificity for cleaving the intracellular signaling molecule c-di-GMP [25], *ycfR*/*bhsA* that encodes a protein involved in biofilm formation through its ability to modulate quorum sensing indole concentrations, multiple stress responses, cell aggregation, and cell surface hydrophobicity [26], and *bdm/yddX*, which encodes a protein involved in biofilm dependent modulation and is regulated by the Rcs two-component system [27]. These findings are quite surprising given that the conditions under which L-form colonies are produced (formed with antibiotics) and biofilms are produced (formed without antibiotics) are quite distinct. However, the typical “fried egg” L-form colony exhibits signs of group behavior similar to that of biofilms, which could explain why they share certain stress pathways in common.

Utilization of the *E. coli* deletion mutant library allowed us to further examine genes required for L-form formation and survival identified by microarray analysis. It is reassuring that most genes identified in the mutant screens were also found to be differentially expressed by microarray analysis. We identified 3 groups of mutants that showed varying degrees of defects in L-form colony formation, possibly reflecting the relative importance of the genes involved in L-form formation (**Fig. 3**), with the majority of the mutants belonging to Group 1 mutants which showed a complete inability to form L-form colonies. Among the 24 Group 1 mutants, only 4 mutants, *acrA, acrB, yfgL*, and *mrcB* mutants, have previously been reported to be hypersensitive to beta-lactam antibiotics [28]. It is possible that their inability to develop L-form colonies could be due to hypersensitivity to penicillin. However, most beta-lactam hypersensitive mutants such as *rpmF, rimK, rplA, pgmB, dksA, phoP, surA, emtA,* identified in a previous study using the same Keio mutant library [28] were able to form L-form colonies in this study. This suggests that increased antibiotic susceptibility under normal MIC testing conditions for classical forms may not necessarily translate into defects in L-form colony formation. However, the majority of the Group 1 mutants, i.e., the remaining 20 mutants are genuine and their lack of L-formation is not due to hypersensitivity to penicillin, but due to a defect specific to L-form formation or survival. Currently, we do not know if the Group 1 mutants have defects in L-form formation or survival. Future studies are needed to determine which step the mutants are defective in.

To confirm that the identified genes in Group 1 mutants are indeed responsible for the defect in L-form formation, we chose 4 representative mutants for complementation studies. These 4 mutants, which represent key pathways identified in the mutant screens, are involved in colanic acid synthesis (*rcsB*), iron homeostasis (*fur*), DNA repair/SOS response (*ruvA*), and a small lipoprotein component of the outer membrane assembly complex (*smpA*). Our initial attempt to complement these mutants with their corresponding wild type gene on a multicopy plasmid vector was unsuccessful, presumably because of the toxicity of uncontrolled overexpression of these gene products. However, using the inducible pBAD vector, we were able to successfully restore the L-form colony formation phenotype of the 4 mutants (**Fig. 4**).

Colonic acid synthesis appears to be essential for L-form colony formation and is regulated by the Rcs phosphorelay system, however, our microarray and mutant screen results suggest that the Rcs phosphorelay system plays a greater role. This is consistent with a recent report which suggests that the Rcs system senses damage to the peptidoglycan layer and contributes to low-level intrinsic beta-lactam antibiotic resistance, which is independent of colanic acid capsule synthesis [29]. The Rcs stress system regulates colanic acid synthesis genes in addition to genes involved in osmoregulation/multiple stress responses, cell division, flagella, motility, chemotaxis, and translational regulation of the *rpoS* sigma factor [27], [30]–[33]. Many of the genes regulated by the Rcs phosphorelay system, as well as the Rcs system itself, were upregulated in our L-form colonies, indicating that this system may be activated and utilized for L-form formation or survival (**Fig. 5** **, Table S1**). This may explain why different *rcs* mutants, *rcsC, rcsB, rcsF* mutants, were unable to form L-form colonies (**Fig. 3** **,** **Fig. 5**).

Hydroxyl radical induced damage of proteins, lipids, and DNA has recently been proposed as an underlying common mechanism of killing for several antibiotics, including beta-lactams [34]. Key to the activation of this killing mechanism is the reaction of reactive oxygen species with intracellular iron from damaged iron-sulfur clusters, resulting in the formation of hydroxyl radicals via the Fenton reaction, which leads to subsequent cell death. Our results suggest that L-forms rely on increased iron sulfur cluster biosynthesis and repair mechanisms to manage the amount of iron available to undergo the Fenton reaction. This is apparent by the up-regulation of several genes within the *isc* gene cluster involved in iron sulfur cluster biosynthesis and repair in our microarray analysis [35](**Table S1**). The ferric uptake regulator (Fur) also appears to be essential for the formation of L-form colonies (**Fig. 4**). Fur is a repressor of iron uptake and regulates iron levels through its ability to control intracellular free iron concentrations, thus minimizing iron-induced redox stresses [36]. Loss of Fur function in the *fur* mutant may lead to increased toxic levels of intracellular iron that can cause excessive reactive oxygen radical production and DNA damage which is not conducive to L-form formation.

DNA repair pathways in response to DNA damage caused by reactive oxygen radicals also appear to be important for L-form colony formation, since maintenance of intact DNA structure is required for replication and viability. The SOS response, which represents one of the DNA repair pathways we identified in the microarray, may also contribute to the delay of L-form colony formation, due to inhibition of cell division during the DNA repair process [37](**Fig. 4**). RuvA is part of a protein complex RuvAB that resolves Holliday junctions and is essential for recombinational repair of DNA lesions, whereas RecG is a DNA translocase that also catalyses branch migration of Holliday junctions like RuvAB but through a different mechanism [38]. It is significant that our mutant screens identified two Group 1 mutants *ruvA* and *recG* that are involved in DNA repair that had a defect in L-form formation or survival (**Fig. 3**).

The small membrane lipoprotein (SmpA) is a non-essential member of the YaeT complex involved in outer membrane biogenesis in Gram-negative bacteria [39]. Along with *smpA,* fellow lipoprotein *yfgL* was also among our Group 1 mutants and was overexpressed in L-form colonies along with fellow lipoprotein members *yfiO*, *nlpB,* and *yfgL.* Although *yfgL* mutants are hypersensitive to ampicillin, *smpA* mutants have not been shown to be hypersensitive. This difference is possibly due to the interaction YfgL has with YaeT, which is independent of the other lipoproteins in the complex. *smpA* mutants are viable and are reported to have only mild defects in their outer membrane [39]. The fact that we did not isolate other mutants with cell envelope defects such as those from the Tat or Tol-Pal systems suggests that inability of the *smpA* mutant to grow on LIM media may not be directly related to its outer membrane defect. This is perhaps due to a defect in initial cell aggregation resulting from changes in its cell surface properties, which affected L-form formation. Further studies are required to test this hypothesis.

Despite the significant findings of this study there are several limitations. Firstly, microarray analysis was performed using 72 hr unstable L-form colonies that had already formed. Thus the array data may have favored identification of genes required for the survival rather than the formation of L-form colonies. This could also explain why the vast majority of significant differentially expressed genes were up-regulated compared to down-regulated. A more comprehensive, temporal microarray analysis over a range of time points may shed more light on the gene expression changes that occur in early stages of L-form colony formation. Secondly, the *E. coli* strain used for the microarray analysis was the W3110 strain which is not the same as the BW25113 strain used in the construction of the deletion mutant library. Although this might cause some minor discrepancies in the correlation between the microarray and the mutant screen data, this should not alter the major findings and conclusions of the study. The fact that the mutant screen data which is more reliable than the microarray findings correlate well with the microarray data suggests that the above limitation is not a major concern. Thirdly, because induction of classical bacteria into L-forms usually require high concentrations of antibiotics, equivalent drug concentration controls and exposure times for classical cells are not possible. Furthermore, our microarray analysis may have included gene expression changes that are the result of cell wall loss and may not necessarily be required for the creation or maintenance of L-forms per se. Even though these gene expression changes may not be specific for L-forms, the data highlights important biological processes that depend on the existence of an intact cell wall. Future microarray analyses utilizing additional controls such as cells grown at a lower concentration of Penicillin G, and cells that are enzymatically stripped of their cell wall might be useful to better pinpoint L-form specific responses. Lastly, microarray analysis and mutant screens used in this study can only examine alterations at the RNA and DNA level that influence L-form formation and survival. Future studies utilizing proteomic and epigenetic analyses will be needed to further investigate changes at the protein level underlying L-form formation and survival.

In conclusion, we report the use of whole genome transcriptome analysis, mutant library screens, and complementation experiments to address the molecular basis of L-form formation. Microarray analysis revealed a network of stress responses and pathways that possibly contribute to the survival of L-form colonies and that are also overexpressed or important in persisters and biofilms. Mutant library screens identified three groups of mutants with varying degrees of defects in L-form formation or survival. Complementation experiments allowed us to confirm four Group 1 mutants, *rcsB*, *ruvA*, *fur*, and *smpA* that are involved in the formation or survival of L-form colonies. This work represents the first systematic genetic study to identify genes and pathways involved in the formation and survival of L-forms. These results shed new insights into the molecular mechanisms underlying L-form formation and survival while also establishing the framework for future research on how the identified pathways and genes interact leading to the emergent properties of L-forms. Our findings have implications for understanding the emergence of antibiotic resistance and bacterial persistence and designing novel drugs and vaccines targeting L-form bacteria for improved control of persistent bacterial infections.

## Materials and Methods

### Culture media and growth conditions

Routine growth of *E. coli* strains was conducted using Luria-Bertani (LB) medium. All bacterial cultures were incubated aerobically at 37°C unless otherwise specified. Kanamycin (50 µg/ml) and chloramphenicol (30 µg/ml) were used in the culture of the mutant library and in the complementation of the mutants, respectively.

### Microscopy

L-form colonies were examined for typical “fried egg” colony morphology using a Nikon GM3 inverted microscope. Hoffman modulation was used for gray scale photos of colonies. Photos were processed using SPOT software. Transmission electron microscopy and freeze substitution of L-form colonies was performed as described [40].

### Induction of L-form colonies

*E. coli* K-12 W3110 [F^−^ *mcrAmcrB* IN (*rrnD*-*rrnE)1* lambda^−^] or *E. coli* K-12 BW25113 [*rrnB3 ΔlacZ4787 hsdR514Δ(araBAD)567 Δ(rhaBAD)568 rph-1*] was grown in LB broth to log phase or overnight to stationary phase. Undiluted along with serial tenfold dilutions of cells were spread (100 µl) or spotted (10 µl) onto L-form induction media (LIM) which consisted of brain heart infusion broth (BHI) Becton Dickinson (BD) supplemented with 1% agar (BD), 10% sucrose, 0.125% MgSO_4_, and 6000 µg (10,000 units )/ml of Penicillin G (Sigma). After the inoculum was absorbed, plates were flipped and incubated aerobically at 37°C. After 48–72 hrs typical “fried egg” appearing L-form colonies grew which contained coccoid cells.

### Microarray procedure and data analysis

To determine the gene expression profile of L-forms, isolated colonies of *E. coli* W3110 were grown to log phase, diluted, and spread onto three culture conditions consisting of (BHI, BHI+Sucrose+MgSO4, BHI+Sucrose+MgSO4+Penicillin G). Triplicate plates were cultured for each condition, resulting in a total of 9 samples from the three conditions for microarray processing. All isolated colonies on each media plate were harvested using a sterile cotton swab saturated with PBS buffer followed by immersion into 500 µl of PBS buffer. Cells were spun for 5 minutes at 8000 rpm to pellet cells using a Microfuge 18 benchtop centrifuge (Beckman Coulter). Supernatant was removed, and the resulting cells were snap-frozen in liquid nitrogen and stored at −80°C. RNA extraction, quality assessment, and processing of samples for gene expression analysis by microarray were performed at the Johns Hopkins Malaria Research Institute Gene Array Core Facility (JHMRI-GACF), using standard Core protocols. Briefly, RNA was extracted using the Master Pure RNA Purification Kit (Epicentre), according to the manufacturer's recommended protocol. Triplicate RNA samples for each condition were processed in accordance with methods described in the Ambion MessageAmp™ II- Bacteria, Prokaryotic RNA Amplification Kit Manual, with the only modification being the use of biotin labeled UTP. Fragmentation of cRNAs and hybridization to Affymetrix *E. coli* Genome 2.0 GeneChips was performed using Affymetrix standard protocols. The signal amplification protocol for washing and staining of prokaryotic targets was performed in an automated fluidics station (Mini_prok2v1, Affymetrix FS450) as described in the Affymetrix Technical Manual, Revision Five. The arrays were transferred to the GCS3000 laser scanner (Affymetrix) and scanned at an emission wavelength of 570 nm at 2.5 µm resolution. For more detailed methods, please refer to the website of the MRI-GACF at the Johns Hopkins Bloomberg School of Public Health http://jhmmi.jhsph.edu/.)

Hybridization intensity raw data - *. CEL files were uploaded to the R-Project Bioconductor statistical tools package [19]. Normalized gene expression values were generated for each array chip by RMA method in Bioconductor Affy package. The quantile normalization forced all probe intensities to conform to the same distribution for each array crossing all 9 arrays. After normalization, we retrieved 5,255 K12 strain probe sets based on Affymetrix *E. coli-*2 Annotation file. We then applied Bioconductor ‘limma’ package software which utilizes linear modeling to perform moderated t-statistic for computing fold changes and adjusted p-values for each gene and each comparison between groups. Benjamini and Hochberg's method was used in order to control the false discover rate [41]. We chose to use the log-odds value which represents the probability of differential expression along with fold change as a double filter for obtaining our final significant differential expressed gene list. Functions, classification, and enriched pathway identification of differentially expressed genes was conducted using the EcoCyc database (http://ecocyc.org/) along with the COG functional classification system. The microarray data have been deposited in the NCBI Gene Expression Omnibus (http://ncbi.nlm.nih.gov/geo) and are accessible through Gene Expression Omnibus series accession number GSE14796.

### SYBR Green real-time PCR

Gene expression of 11 up-regulated genes from different pathways was verified using a Quantitative real-time SYBR Green MasterMix Kit (Applied Biosystems) on an Applied Biosystems 7300 real-time instrument and ABI Prism SDS 1.2.2. software. Primers corresponding to the 11 genes of interest were designed using Primer Express software (version 2.0, Applied Biosystems). Standardized total RNA was converted to cDNA using SuperScript III First-Strand Synthesis (Invitrogen) as described by the manufacturer. cDNA was then used as template to perform real-time PCR following Applied Biosystems protocols. The 16S rRNA gene was used as the reference gene for comparison with the genes of interest. Changes of expression are the average of three biological replicates.

### Library screen for mutants defective in L-form colony formation

The Keio collection, a non-essential gene library consisting of 3985 single-gene deletion mutants of *E. coli* K-12 BW25113 [42], was grown at 37°C overnight in 100 µl of LB medium in 96-well plates without shaking. Log phase, as well as stationary phase cultures, were replica transferred onto LB plates as a growth control and LIM plates. Plates were allowed to dry before being inverted and incubated at 37°C for up to 72 hrs before mutants were scored for defects in forming L-form colonies.

### Complementation of L-form mutants

Complementation of deletion mutants (*ruvA, rcsB, fur, smpA*) was performed utilizing the arabinose inducible low copy vector pBAD33-cmr [43]. A functional wild type copy of each gene was amplified along with the optimized SD sequence (AGGAGG) incorporated into PCR primers. PCR products were digested with restriction enzymes *Eco*RI and *Hind*III and cloned into pBAD33 using the quick ligation kit by New England Biolabs. The resulting constructs were transformed along with the empty pBAD33 vector into each mutants as well as the BW25113 parent strain. Overnight cultures of transformed mutants and parent strain were spotted onto LIM with and without 0.2% arabinose. Plates were observed after 72 hr incubation at 37°C for any growth of typical L-form colonies macroscopically and microscopically.

## Supporting Information

Table S1 Genes overexpressed 4-fold and higher in E. coli L-form colonies versus classical colonies
(0.04 MB PDF)

Table S2 Genes repressed 4-fold and higher in E. coli L-form colonies versus classical colonies
(0.01 MB PDF)

Table S3 Correlation of microarray data with real-time PCR results
(0.03 MB DOC)

Table S4 Primers used for real-time PCR
(0.03 MB DOC)
