# Supplementary material for: Insights into the Molecular Basis of L-Form Formation and Survival in Escherichia coli
Source: PLoS One. 2009 Oct 6;4(10):e7316. doi: 10.1371/journal.pone.0007316 (PMC2752164; doi:10.1371/journal.pone.0007316)
Supplement: Table S1 — Genes overexpressed 4-fold and higher in E. coli L-form colonies versus classical colonies (0.04 MB PDF) [file pone.0007316.s001.pdf]

Table S1. Genes overexpressed 4-fold and higher in *E. coli* L-form colonies versus classical colonies

Fold change	Gene	Product-function
**1. INFORMATION STORAGE AND PROCESSING**		
**DNA replication, recombination and repair**		
48.2	*recN*	recombination and repair protein
46.4	*dinD*	DNA-damage-inducible protein
44.7	*yebG*	DNA damage-inducible protein YebG
27.4	*recA*	DNA strand exchange and recombination protein
26.4	*recX*	recombination regulator RecX
18.2	*ruvB*	Holliday junction DNA helicase B
17.2	*ruvA*	Holliday junction DNA helicase motor protein
17.1	*dinI*	DNA damage-inducible protein I
16.4	*obgE*	GTPase ObgE
14.8	*yqgF*	Holliday junction resolvase-like protein
11.2	*umuD*	DNA polymerase V subunit UmuD
10.5	*lexA*	LexA repressor
9.2	*mcrA*	type IV site-specific deoxyribonuclease/DNA
8.9	*iscR*	DNA-binding transcriptional regulator
8.3	*holD*	DNA polymerase III subunit psi
8.3	*rdgC*	recombination associated protein
7.8	*topA*	DNA topoisomerase I
7.5	*uvrD*	DNA-dependent helicase II
7.2	*deoR*	DNA-binding transcriptional repressor
6.8	*yfgE*	DNA replication initiation factor
6.8	*ligA*	NAD-dependent DNA ligase LigA
5.7	*seqA*	replication initiation regulator SeqA
5.0	*holB*	DNA polymerase III subunit delta'
4.9	*dnaX*	DNA polymerase III subunits gamma and tau
**Translation, ribosomal structure and metabolism**		
32.2	*rplW*	50S ribosomal protein L23
31.5	*infB*	translation initiation factor IF-2
29.5	*isrB*	small RNA
27.3	*truB*	tRNA pseudouridine synthase B
24.1	*rplI*	50S ribosomal protein L9
18.6	*rplD*	50S ribosomal protein L4
14.1	*yhdG*	tRNA-dihydrouridine synthase B
14.1	*alaS*	subunit of alanyl-tRNA synthase
12.9	*yhbC*	ribosomal maturation protein
12.8	*rbfA*	ribosome-binding factor A
11.2	*hisS*	histidyl-tRNA synthetase
10.3	*ycbY*	23S rRNA m^2G2445 methyltransferase
9.8	*ykgM*	50S ribosomal protein L31 type B
9.8	*leuS*	leucyl-tRNA synthetase
9.2	*gidA*	tRNA uridine 5-carboxymethylaminomethyl enzyme
8.4	*rplY*	50S ribosomal protein L25
7.6	*rsuA*	16S rRNA pseudouridylate synthase A
7.3	*engA*	50s ribosomal subunit stability factor
6.9	*glyQ*	glycyl-tRNA synthetase subunit alpha
6.9	*glnS*	glutaminyl-tRNA synthetase

6.5	*yjbN*	tRNA-dihydrouridine synthase A
6.4	*gltX*	glutamyl-tRNA synthetase
6.3	*lysS*	lysyl-tRNA synthetase
6.1	*prfB*	peptide release factor RF-2
5.9	*selD*	selenide, water dikinase
5.7	*serS*	seryl-tRNA synthetase

Transcription

26.5	*emrR*	transcriptional repressor MprA
26.2	*cspA*	transcriptional activator cold shock protein
22.8	*stpA*	DNA binding protein, nucleoid-associated
22.1	*pdhR*	transcriptional regulator PdhR
18.2	*ilvY*	DNA-binding transcriptional regulator
16.2	*rpoD*	sigma D factor
15.6	*marA*	DNA-binding transcriptional activator MarA
14.2	*deaD*	ATP-dependent RNA helicase DeaD
13.9	*cbl*	transcriptional regulator Cbl
13.8	*narP*	DNA-binding response regulator
13.6	*soxS*	DNA-binding transcriptional dual regulator
12.4	*gcvA*	DNA-binding transcriptional activator GcvA
12.4	*evgA*	DNA-binding response regulator in two-component
12.4	*rfaH*	transcriptional activator RfaH
12.1	*rcsB*	DNA-binding response regulator in two-component
12.1	*ycjC*	DNA-binding transcriptional repressor
11.1	*nusB*	transcription antitermination protein NusB
10.3	*mngR*	DNA-binding transcriptional dual regulator, fatty-acyl-binding
10.2	*yhiX*	DNA-binding transcriptional dual regulator
10.0	*bglJ*	DNA-binding transcriptional activator BglJ
9.9	*phoB*	DNA-binding response regulator in two-component
9.4	*xapR*	DNA-binding transcriptional activator
9.4	*rho*	transcription termination factor Rho
9.3	*yaiN*	regulator protein FrmR
9.0	*greA*	transcription elongation factor GreA
8.3	*zntR*	zinc-responsive transcriptional regulator
8.1	*pspC*	DNA-binding transcriptional activator
8.0	*glnG*	nitrogen regulation protein NR(I)
7.9	*sdiA*	DNA-binding transcriptional activator
7.7	*marR*	DNA-binding transcriptional repressor MarR
7.5	*fimZ*	transcriptional regulator FimZ
6.7	*yohL*	RcnR transcriptional repressor
6.4	*yciT*	transcriptional regulator
5.7	*bglG*	transcriptional antiterminator BglG
5.6	*fimB*	tyrosine recombinase/inversion of on/off regulator of fimA
5.6	*nhaR*	transcriptional activator NhaR

2. CELLULAR PROCESSES

Adaptation, defense and survival

59.7	*pspB*	phage shock protein B
53.4	*rcsA*	colanic acid capsular biosynthesis activation protein A
51.4	*pspG*	phage shock protein G
41.1	*pspA*	phage shock protein A

33.9	*wza*	capsular polysaccharide transport
32.0	*wcaF*	putative colanic acid biosynthesis acetyltransferase WcaF
29.8	*wcaH*	GDP-mannose mannosyl hydrolase
28.7	*gmd*	GDP-mannose 4,6-dehydratase
21.6	*ycfR*	stress resistance/biofilm formation
20.9	*cspG*	cold-shock
19.8	*wcaD*	putative colanic acid polymerase
17.9	*ugd*	UDP-glucose 6-dehydrogenase
17.5	*pspD*	peripheral inner membrane phage-shock protein
17.1	*chpA*	toxin ChpA
15.9	*wzb*	tyrosine phosphatase/colanic acid
15.2	*ykfE*	vertebrate C-lysozyme inhibitor
14.6	*marB*	multiple antibioitc resistance protein
12.1	*chpR*	antitoxin MazE
10.0	*acrB*	RND-permease/drug efflux
9.5	*acrA*	membrane fusion protein/drug efflux system
9.4	*cpsB*	mannose-1-phosphate guanylyltransferase
8.9	*yaiM*	serine hydrolase
8.4	*yfiD*	autonomous glycyl radical cofactor GrcA
7.9	*osmB*	lipoprotein
7.4	*mazG*	nucleoside triphosphate pyrophosphohydrolase
6.6	*ybaU*	peptidyl-prolyl cis-trans isomerase (rotamase D)
5.5	*manA*	mannose-6-phosphate isomerase
5.0	*cpsG*	Phosphomannomutase
4.8	*galU*	UTP--glucose-1-phosphate uridylyltransferase subunit GalU
4.7	*chpS*	antitoxin

Post-translational modification, protein turnover and chaperones

43.5	*fhuF*	ferric iron reductase involved in ferric hydroximate transport
41.3	*yjiD*	inhibitor of sigma factor s proteolysis
27.2	*lspA*	lipoprotein signal peptidase
16.7	*ompT*	outer membrane protease
15.9	*hslJ*	heat-inducible protein
15.9	*ytfE*	repair of damaged iron-sulfur clusters
13.7	*ppiC*	peptidyl-prolyl cis-trans isomerase C
12.6	*yrfH*	ribosome-associated heat shock protein Hsp15
12.5	*iscU*	scaffold protein
10.9	*dsbA*	periplasmic protein disulfide isomerase I
10.5	*pepB*	aminopeptidase B
10.4	*groEL*	chaperonin GroEL
9.7	*hflC*	FtsH protease regulator HflC
8.8	*hslV*	ATP-dependent protease peptidase subunit
8.7	*ibpB*	heat shock chaperone IbpB
7.8	*iap*	aminopeptidase in alkaline phosphatase isozyme conversion
7.7	*fkpA*	FKBP-type peptidyl-prolyl cis-trans isomerase (rotamase)
7.0	*hscB*	co-chaperone HscB
7.0	*pphB*	serine/threonine-specific protein phosphatase 2
6.7	*dsbC*	thiol:disulfide interchange protein DsbC
6.2	*ffh*	signal recognition particle protein
5.9	*ybbN*	chaperone and protein oxidoreductase
4.9	*dcp*	dipeptidyl carboxypeptidase II

4.8	*dnaJ*	chaperone protein DnaJ
4.3	*htpG*	heat shock protein 90
**Cell envelope biogenesis**		
27.1	*cld*	regulator of length of O-antigen component
21.7	*rfbD*	dTDP-4-dehydrorhamnose reductase subunit,
17.2	*yidC*	putative inner membrane protein translocase component
15.1	*rfbB*	dTDP-glucose 4,6 dehydratase, NAD(P)-binding
14.2	*nlpA*	cytoplasmic membrane lipoprotein-28
14.0	*amiC*	N-acetylmuramoyl-L-alanine amidase amiC precursor
13.2	*ispH*	4-hydroxy-3-methylbut-2-enyl diphosphate reductase
9.9	*rfbA*	glucose-1-phosphate thymidylyltransferase
9.1	*yaeS*	undecaprenyl pyrophosphate synthase
8.9	*ddg*	lipid A biosynthesis palmitoleoyl acyltransferase
8.5	*kdsA*	2-dehydro-3-deoxyphosphooctonate aldolase
8.3	*rfaP*	kinase that phosphorylates core heptose of lipopolysaccharide
8.0	*rfaD*	ADP-L-glycero-D-mannoheptose-6-epimerase
7.1	*wecB*	UDP-N-acetylglucosamine 2-epimerase
7.0	*rfaG*	glucosyltransferase I
6.3	*mreC*	cell wall structural complex MreBCD
6.0	*wbbI*	β-1,6-galactofuranosyltransferase
5.6	*rfaJ*	(galactosyl)lipopolysaccharide glucosyltransferase
5.5	*rfaQ*	lipopolysaccharide core biosynthesis protein
5.2	*murI*	glutamate racemase
4.8	*yrbI*	3-deoxy-D-manno-octulosonate 8-phosphate phosphatase
**Transport**		
39.2	*sbp*	sulfate transporter subunit
21.5	*cysP*	thiosulfate transporter subunit
21.1	*pstS*	phosphate transporter subunit
18.7	*efeU*	putative cytochrome
13.7	*exbD*	biopolymer transport protein ExbD
11.9	*emrA*	multidrug resistance protein A
11.3	*phoU*	transcriptional regulator PhoU
11.2	*kgtP*	alpha-ketoglutarate transporter
10.5	*oppA*	periplasmic oligopeptide-binding protein precursor
8.6	*pstB*	phosphate transporter subunit
8.6	*proV*	glycine betaine transporter ATP-binding subunit
8.3	*secE*	preprotein translocase subunit SecE
8.1	*lolA*	outer-membrane lipoprotein carrier protein
8.0	*proX*	glycine betaine transporter periplasmic subunit
7.5	*fliY*	cystine transporter subunit
6.3	*tatE*	twin arginine translocase protein E
5.6	*cysW*	sulfate/thiosulfate transporter permease subunit
**Cell division and chromosomal partitioning**		
29.5	*sulA*	SOS cell division inhibitor
13.6	*hflK*	FtsH protease regulator HflK
10.1	*ftsY*	cell division protein FtsY
6.9	*tig*	trigger factor
6.8	*xerC*	site-specific tyrosine recombinase XerC

6.7	*ftsN*	essential cell division protein
5.4	*yhjQ*	cell division protein

Inorganic ion transport and metabolism

16.6	*cirA*	ferric iron-catecholate outer membrane transporter
10.4	*corA*	magnesium/nickel/cobalt transporter CorA
9.3	*copA*	copper transporter
8.7	*znuA*	high-affinity zinc transporter periplasmic component
8.5	*znuC*	high-affinity zinc transporter ATPase
5.3	*fepB*	iron-enterobactin transporter periplasmic binding protein

Signal transduction mechanism

8.7	*phoR*	phosphate regulon sensor protein
7.3	*rcsC*	hybrid sensory kinase in two-component

Protein assembly

12.8	*iscA*	iron-sulfur cluster assembly protein

Motility and chemotaxis

20.2	*ycdT*	diguanylate cyclase

3. METABOLISM

Amino acid transport and metabolism

34.3	*cysK*	cysteine synthase A
17.7	*tyrA*	bifunctional chorismate mutase/prephenate dehydrogenase
16.4	*gltP*	glutamate/aspartate:proton symporter
11.7	*ilvG*	acetolactate synthase 2 catalytic subunit
11.7	*metQ*	DL-methionine transporter substrate-binding subunit
10.7	*avtA*	valine--pyruvate transaminase
10.3	*aroB*	3-dehydroquinate synthase
8.8	*iscS*	cysteine desulfurase
8.7	*argG*	argininosuccinate synthase
8.3	*glnA*	glutamine synthetase
7.9	*proB*	gamma-glutamyl kinase
6.5	*yedO*	D-cysteine desulfhydrase
6.0	*thrC*	threonine synthase
5.7	*metK*	methionine adenosyltransferase
5.6	*ybiK*	L-asparaginase

Carbohydrate transport and metabolism

14.6	*ybiV*	sugar phosphatase
14.6	*lpdA*	dihydrolipoamide dehydrogenase
11.8	*yadF*	carbonic anhydrase
10.0	*glpD*	glycerol-3-phosphate dehydrogenase
9.8	*gnd*	6-phosphogluconate dehydrogenase
7.4	*ppsA*	phosphoenolpyruvate synthase
6.3	*adhC*	Alcohol dehydrogenase class III
6.3	*eda*	keto-hydroxyglutarate-aldolase
5.5	*fruB*	fructose-specific PTS IIA/HPr components
5.2	*yfbT*	sugar phosphatase
4.9	*zwf*	glucose-6-phosphate 1-dehydrogenase

4.5	*ackA*	acetate kinase

Energy production and conversion

25.4	*acnB*	bifunctional aconitate hydratase 2/2-methylisocitrate
19.6	*aceE*	pyruvate dehydrogenase subunit E1
19.1	*nudE*	ADP-ribose diphosphatase NudE
16.7	*aceF*	dihydrolipoamide acetyltransferase
16.6	*cysJ*	sulfite reductase subunit alpha
15.0	*cysC*	adenylylsulfate kinase
14.6	*cysM*	cysteine synthase B
14.1	*csdA*	cysteine sulfinate desulfinase
14.0	*cysH*	phosphoadenosine phosphosulfate reductase
11.3	*yecK*	Cytochrome c-type protein torY
11.0	*fdx*	Ferredoxin, 2Fe-2S
9.5	*yjhT*	N-acetylneuraminate mutarotase
9.4	*ldhA*	D-lactate dehydrogenase
8.8	*ubiE*	ubiquinone/menaquinone biosynthesis methyltransferase
8.4	*glpE*	thiosulfate sulfurtransferase
6.2	*ahpF*	Alkyl hydroperoxide reductase subunit F
6.0	*ndh*	respiratory NADH dehydrogenase
5.9	*yojH*	malate:quinone oxidoreductase
5.4	*cysN*	sulfate adenylyltransferase, subunit 1

Nucleotide transport, conversions and metabolism

33.0	*yahA*	c-di-GMP
12.7	*gpt*	xanthine-guanine phosphoribosyltransferase
9.6	*adk*	adenylate kinase
8.8	*gmk*	guanylate kinase
8.3	*pyrG*	CTP synthase
7.5	*nrdI*	ribonucleotide reductase stimulatory protein
7.1	*tmk*	thymidylate kinase
5.9	*deoB*	phosphopentomutase
5.8	*tdk*	thymidine kinase
4.6	*nrdE*	ribonucleotide-diphosphate reductase subunit alpha
13.6	*cyaA*	adenylate cyclase
7.7	*purU*	formyltetrahydrofolate deformylase
5.7	*yjjG*	nucleotidase
5.6	*yfbR*	dCMP phosphohydrolase

Cofactor and coenzyme metabolism

12.4	*cobT*	nicotinate-nucleotide- phosphoribosyltransferase
9.4	*mioC*	flavodoxin
11.4	*hemA*	glutamyl-tRNA reductase
8.1	*ribH*	riboflavin synthase subunit beta
7.9	*yaeM*	1-deoxy-D-xylulose 5-phosphate reductoisomerase
7.0	*ribB*	3,4-dihydroxy-2-butanone 4-phosphate synthase
5.9	*ribF*	bifunctional riboflavin kinase/FMN adenylyltransferase
5.8	*pncB*	nicotinate phosphoribosyltransferase
5.0	*pabC*	4-amino-4-deoxychorismate lyase

Lipid metabolism

18.1	*cdh*	CDP-diacylglycerol pyrophosphatase
6.2	*accC*	acetyl-CoA carboxylase biotin carboxylase subunit
5.7	*accB*	acetyl-CoA carboxylase biotin carboxyl carrier subunit

Phosphate metabolism

11.5	*phoA*	bacterial alkaline phosphatase

4. EXTRACHROMOSOMAL

Phage, phage-like element, transposon

138.4	*ymfJ*	e14 prophage; predicted protein
48.3	*xisE*	e14 prophage; predicted excisionase
31.8	*intE*	e14 prophage; predicted integrase
27.3	*pal*	peptidoglycan-associated outer membrane lipoprotein
21.5	*ymfT*	e14 prophage; predicted DNA-binding transcriptional regulator
19.0	*intG*	hypothetical protein
18.7	*insK*	IS150 conserved protein InsB
16.4	*insJ*	IS150 protein InsA
16.3	*ybcY*	hypothetical protein
16.2	*ymfM*	e14 prophage; predicted protein
14.3	*yagK*	CP4-6 prophage; conserved protein
13.3	*ymfD*	e14 prophage; predicted SAM-dependent methyltransferase
10.2	*yjhC*	KpLE2 phage-like element; predicted oxidoreductase
9.7	*ymfL*	e14 prophage; predicted DNA-binding transcriptional regulator
8.9	*yfdI*	CPS-53 (KpLE1) prophage; predicted inner membrane protein
8.2	*insN-2*	KpLE2 phage-like element
7.7	*ybcW*	prophage
6.9	*ybcM*	DLP12 prophage DNA-binding transcriptional regulator
6.3	*yfdH*	CPS-53 (KpLE1) prophage; bactoprenol glucosyl transferase
6.1	*insB-7*	IS1 protein InsB
5.6	*yjhI*	KpLE2 phage-like element
5.4	*yfjO*	CP4-57 prophage; predicted protein

5. UNKNOWN AND POORLY CHARACTERIZED

Conserved proteins

26.3	*yafO*	putative toxin YafO
24.6	*ybjX*	hypothetical protein ybjX
22.8	*yhhI*	hypothetical protein
21.1	*yebE*	hypothetical protein yebE
20.7	*yafK*	hypothetical protein yafK precursor
19.2	*yhcN*	hypothetical protein yhcN precursor
17.1	*yeeD*	hypothetical protein
16.2	*yjaA*	hypothetical protein
14.5	*yfeS*	conserved protein
14.2	*ygjN*	hypothetical protein
11.9	*yqiC*	conserved protein
11.1	*ygdL*	hypothetical protein ygdL
10.8	*yibG*	hypothetical protein
10.4	*ycbK*	hypothetical protein ycbK
9.9	*yhdN*	hypothetical protein
8.9	*yabB*	hypothetical protein
8.9	*ygbE*	hypothetical protein

7.7	*yjiV*	pseudogene
7.5	*yjeO*	conserved inner membrane protein
6.6	*yhgI*	hypothetical protein
6.2	*matB*	hypothetical protein
5.7	*aroM*	hypothetical protein
5.4	*yggE*	hypothetical protein
5.3	*ybaB*	hypothetical protein
5.0	*ycaQ*	hypothetical protein ycaQ
4.8	*yhfY*	hypothetical protein yhfY
**Predicted function**		
35.7	*iscX*	hypothetical protein
23.4	*smpA*	small membrane protein, lipoprotein
20.9	*yedV*	putative 2-component sensor protein
16.3	*yrfF*	putative membrane protein igaA homolog
16.3	*yeeE*	putative transport system permease protein
16.2	*wcaE*	predicted glycosyl transferase
16.2	*ydjN*	hypothetical symporter ydjN
16.1	*yibD*	predicted glycosyl transferase
16.1	*yqeH*	hypothetical protein
14.6	*yjbF*	hypothetical lipoprotein yjbF precursor
13.6	*yihK*	GTP-binding protein
12.6	*yafN*	putative antitoxin of the YafO-YafN toxin-antitoxin system
12.4	*yhgF*	transcrptional accessory protein
12.3	*ygjM*	hypothetical protein ygjM
11.0	*era*	GTP-binding protein Era
10.3	*ygeV*	hypothetical sigma-54-dependent transcriptional regulator
10.2	*ygiS*	putative binding protein
9.3	*ycbL*	hypothetical protein ycbL
9.3	*rfbX*	predicted polisoprenol-linked O-antigen transporter
9.3	*yaeR*	hypothetical protein
9.2	*ybeX*	Magnesium and cobalt efflux protein corC
9.0	*bdm*	biofilm-dependent modulation protein
9.0	*ybiS*	hypothetical protein
8.5	*yjgM*	hypothetical protein
8.3	*yedW*	transcriptional regulatory protein YedW
8.2	*yfgL*	outer membrane protein assembly complex subunit YfgL
7.8	*yajO*	putative NAD(P)H-dependent xylose reductase
7.8	*eco*	ecotin precursor
7.6	*yebA*	hypothetical protein
7.2	*ybdG*	hypothetical protein ybdG
7.1	*yfjG*	hypothetical protein
6.8	*yhjX*	major facilitator superfamily transporter
6.4	*ybgF*	hypothetical protein
6.3	*yfiO*	predicted lipoprotein
6.2	*yrbD*	hypothetical protein
6.0	*vacB*	Rnase R
6.0	*yrbK*	hypothetical protein
6.0	*damX*	hypothetical protein
6.0	*ycfQ*	hypothetical transcriptional regulator
5.9	*sbmA*	transport protein

5.6	*yrbC*	protein yrbC precursor
**Predicted protein**		
43.6	*ypfG*	hypothetical protein ypfG precursor
32.7	*yegZ*	pseudo
28.4	*yqgB*	hypothetical protein yqgB
28.1	*spy*	periplasmic protein, spheroblast
25.5	*ypeC*	hypothetical protein
25.2	*ycfJ*	hypothetical protein
24.7	*yibA*	hypothetical protein
23.0	*ygaC*	hypothetical protein
21.0	*ydhA*	hypothetical protein
20.0	*yajI*	hypothetical protein
18.4	*yfdX*	hypothetical protein
18.0	*htrL*	hypothetical protein
17.1	*yeeN*	hypothetical protein
14.7	*yfgM*	hypothetical protein yfgM
13.8	*ycbW*	hypothetical protein ycbW
13.7	*ylbH*	conserved protein
13.2	*yggN*	hypothetical protein
12.3	*yobH*	hypothetical protein
12.2	*yjcB*	hypothetical protein yjcB
12.1	*yibJ*	rhsA core protein with extension
12.0	*ais*	Ais protein
11.8	*yjjQ*	hypothetical protein yjjQ
11.7	*ysaB*	hypothetical protein
11.6	*yfbP*	predicted protein
11.5	*yedJ*	hypothetical protein
11.3	*wbbL*	pseudo
11.3	*ykiA*	hypothetical protein ykiA
11.0	*yiiF*	conserved protein
11.0	*sseB*	protein sseB
10.9	*matA*	hypothetical protein ykgK
10.8	*ybeB*	hypothetical protein
10.8	*yidX*	predicted lipoproteinC
10.6	*yadM*	predicted fimbrial-like adhesin protein
10.3	*yfjF*	hypothetical protein
10.2	*yejK*	nucleoid-associated protein NdpA
10.0	*yohN*	hypothetical protein yohN precursor
9.7	*wbbJ*	predicted acyl transferase
9.6	*ybbC*	predicted protein
9.3	*yaiY*	hypothetical protein yaiY
9.3	*nlpB*	lipoprotein
9.2	*yjeJ*	hypothetical protein yjeJ
9.0	*wcaI*	predicted glycosyl transferase
9.0	*yfcL*	hypothetical protein yfcL
9.0	*yncE*	hypothetical protein yncE precursor
8.5	*yhhX*	putative oxidoreductase yhhX
8.4	*ygeO*	hypothetical protein
8.3	*ygeK*	hypothetical protein
8.2	*yceB*	predicted lipoprotein

8.1	*syd*	SecY interacting protein Syd
7.9	*yhhH*	predicted protein
7.9	*yfiR*	hypothetical protein yfiR precursor
7.5	*yafE*	hypothetical protein yafE
7.4	*ygeP*	hypothetical protein
7.4	*ygcK*	predicted protein
7.1	*yfgJ*	hypothetical protein
6.8	*yciF*	putative structural protein
6.7	*yfbN*	predicted protein
6.6	*yejG*	hypothetical protein
6.5	*yqeK*	hypothetical protein
6.4	*yeiS*	hypothetical protein
6.4	*ybeA*	hypothetical protein
6.3	*yebY*	hypothetical protein yebY precursor
6.1	*wcaM*	predicted colanic acid biosynthesis protein
5.9	*ynjI*	hypothetical protein
5.6	*yabI*	conserved inner membrane protein
5.4	*yecT*	hypothetical protein
5.0	*yebO*	hypothetical protein yebO
4.8	*yeeY*	hypothetical transcriptional regulator yeeY
4.7	*ybhK*	predicted transferase
