# Supplementary material for: Insights into the Molecular Basis of L-Form Formation and Survival in Escherichia coli
Source: PLoS One. 2009 Oct 6;4(10):e7316. doi: 10.1371/journal.pone.0007316 (PMC2752164; doi:10.1371/journal.pone.0007316)
Supplement: Table S2 — Genes repressed 4-fold and higher in E. coli L-form colonies versus classical colonies (0.01 MB PDF) [file pone.0007316.s002.pdf]

**Table S2.** Genes repressed 4-fold and higher in *E. coli* L-form colonies versus classical colonies

| Fold change | Gene | Product-function |
|---|---|---|
| **1. CELLULAR PROCESSES** | | |
| **Adaptation, defense and survival** | | |
| 0.17 | *speB* | agmatinase |
| | | |
| **Post-translation modification, protein turnover, chaperones** | | |
| 0.24 | *citX* | 2'-(5''-triphosphoribosyl)-3'-dephospho-CoA:apo-citrate lyase |
| | | |
| **Transport** | | |
| 0.15 | *nupC* | nucleoside (except guanosine) transporter |
| | | |
| **Motility and chemotaxis** | | |
| 0.21 | *cheR* | chemotaxis regulator, protein-glutamate methyltransferase |
| 0.17 | *fliM* | flagellar motor switch protein FliM |
| | | |
| **2. METABOLISM** | | |
| **Amino acid transport and metabolism** | | |
| 0.15 | *astB* | succinylarginine dihydrolase |
| | | |
| **Carbohydrate transport and metabolism** | | |
| 0.22 | *araA* | L-arabinose isomerase |
| 0.19 | *treC* | trehalose-6-phosphate hydrolase |
| | | |
| **Nucleotide transport, conversions and metabolism** | | |
| 0.18 | *xdhA* | xanthine dehydrogenase subunit XdhA |
| | | |
| **3. EXTRACHROMOSOMAL** | | |
| **Phage, phage-like element, transposon** | | |
| 0.17 | *yeeL* | pseudogene |
| 0.16 | *yeeV* | CP4-44 prophage |
| 0.08 | *phnE* | pseudogene |
| | | |
| **4. UNKNOWN AND POORLY CHARACTERIZED** | | |
| **Predicted function** | | |
| 0.14 | *ydcS* | putative ABC transporter periplasmic binding protein |
| 0.13 | *phnJ* | component of a carbon-phosphorous lyase enzyme |
| 0.11 | *rdlA* | misc_RNA |
| 0.09 | *phnI* | carbon-phosphorus lyase complex subunit |
| 0.08 | *sokB* | misc_RNA |
| 0.07 | *rydB* | misc_RNA |
| 0.07 | *rdlB* | misc_RNA |
| 0.06 | *rdlC* | misc_RNA |
| | | |
| **Predicted protein** | | |
| 0.15 | *ytfR* | putative ATP-binding component of a transport system |
| 0.13 | *ygfQ* | predicted protein |
| 0.09 | *ychS* | predicted protein |
