# Supplementary material for: Insights into the Molecular Basis of L-Form Formation and Survival in Escherichia coli
Source: PLoS One. 2009 Oct 6;4(10):e7316. doi: 10.1371/journal.pone.0007316 (PMC2752164; doi:10.1371/journal.pone.0007316)
Supplement: Table S3 — Correlation of microarray data with real-time PCR results (0.03 MB DOC) [file pone.0007316.s003.doc]

**Table S3.** Correlation of microarray data with real-time PCR results

| **Gene** | **Microarray**** | **Real-Time PCR**** |
| --- | --- | --- |
| *marA* | 4.0 | 4.0 |
| *spy* | 4.8 | 4.8 |
| *sbp* | 5.2 | 5.2 |
| *phoB* | 3.3 | 3.3 |
| *ycfJ* | 4.6 | 4.7 |
| *ispH* | 3.7 | 3.7 |
| *pspA* | 5.4 | 5.4 |
| *sulA* | 4.9 | 4.9 |
| *recA* | 4.8 | 4.7 |
| *cysK* | 5.1 | 4.8 |
| *ytfE* | 4.0 | 3.9 |

*Log Base 2 of fold change
