# Supplementary material for: Insights into the Molecular Basis of L-Form Formation and Survival in Escherichia coli
Source: PLoS One. 2009 Oct 6;4(10):e7316. doi: 10.1371/journal.pone.0007316 (PMC2752164; doi:10.1371/journal.pone.0007316)
Supplement: Table S4 — Primers used for real-time PCR (0.03 MB DOC) [file pone.0007316.s004.doc]

| **Gene** | **Primer** | **Sequence (5’ to 3’)** |
| --- | --- | --- |
| 16S rRNA | Forward  Reverse | CAAGTCATCATGGCCCTTACG  ACGCACTTTATGAGGTCCGCT |
| *marA* | Forward  Reverse | GGCACCTGCAACGGATGTTTAAAA  CGGCTCGTTACTTTCCTTCAGCTT |
| *spy* | Forward  Reverse | ACTGCACTGTTTGTTGCCTCTACCC  TGCCTTTGTGGTGCATCATCG |
| *sbp* | Forward  Reverse | CGCGCGAATTGTACGAACA TTTACCTGAGCCACCGTGTGA |
| *phoB* | Forward  Reverse | ATCAAACACCTCAAGCGCGA  GCGAAAACGGCTTGGTGATATA |
| *ycfJ* | Forward  Reverse | GGCCAGTCTGAACGTGTTTGAA  ACTGTGACGTTGCGACACTCCT |
| *ispH* | Forward  Reverse | GGTGGTCGGTTCGAAAAACTCCTC  GGATGTCTTTCGCATCGTCAATCA |
| *pspA* | Forward  Reverse | TGGTTCGTCTGATGATCCAGGAGA  GTTCAATACGGCGAGTCAGCTGTTT |
| *sulA* | Forward  Reverse | CACCGCAACAAAAACTGAGTCGG  GCGTAAAGCGCGAACCATTGAC |
| *cysK* | Forward  Reverse | CTTCAGCGTTAAGTGCCGTATCGG  AGGCCAGTGCAATCCCGGTATT |
| *ytfE* | Forward  Reverse | AGCAACTGCCGGAGCTGATTCT  GGAAAGCTCTTCATGCAGCATGGT |
| *recA* | Forward  Reverse | AAAGGTGAGAAGATCGGTCAGG  GCTCAGCAGCAACTCACGTACT |

**Table S4**. Primers used for real-time PCR
